# Assessing the application of human-centered design to translational research

**DOI:** 10.1017/cts.2021.794

**Published:** 2021-05-24

**Authors:** Marie K. Norman, Megan E. Hamm, Yael Schenker, Colleen A. Mayowski, William Hierholzer, Doris M. Rubio, Steven E. Reis

**Affiliations:** 1 Institute for Clinical Research Education, University of Pittsburgh, School of Medicine Pittsburgh, PA, USA; 2 Clinical and Translational Science Institute, University of Pittsburgh; 3 Center for Research in Health Care’s Data Center, University of Pittsburgh; 4 Palliative Research Center (PaRC) and Section of Palliative Care and Medical Ethics, Division of General Internal Medicine, University of Pittsburgh, School of Medicine

**Keywords:** Team science, human centered design, innovation, collaboration, inclusion, creativity

## Abstract

**Introduction:**

Human-centered design (HCD) training offers the potential to improve both team processes and products. However, the use of HCD to improve the quality of team science is a relatively recent application, and its benefits and challenges have not been rigorously evaluated. We conducted a qualitative study with health sciences researchers trained in HCD methods. We aimed to determine how researchers applied HCD methods and perceived the benefits and barriers to using HCD on research teams.

**Methods:**

We conducted 1-hour, semi-structured interviews with trainees from three training cohorts. Interviews focused on perceptions of the training, subsequent uses of HCD, barriers and facilitators, and perceptions of the utility of HCD to science teams. Data analysis was conducted using Braun and Clarke’s process for thematic analysis.

**Results:**

We interviewed nine faculty and nine staff trained in HCD methods and identified four themes encompassing HCD use, benefits, challenges, and tensions between HCD approaches and academic culture.

**Conclusions:**

Trainees found HCD relevant to research teams for stakeholder engagement, research design, project planning, meeting facilitation, and team management. They also described benefits of HCD in five distinct areas: creativity, egalitarianism, structure, efficiency, and visibility. Our data suggest that HCD has the potential to help researchers work more inclusively and collaboratively on interdisciplinary teams and generate more innovative and impactful science. The application of HCD methods is not without challenges; however, we believe these challenges can be overcome with institutional investment.

## Introduction

With the highest impact science now conducted on large, interdisciplinary, and often geographically distributed research teams [1–3], universities and funding agencies have an increased focus on the factors that produce more productive and innovative teams [1,4]. Scholars have observed the need for targeted training to help researchers develop the skills and mindsets necessary to engage effectively in collaborative problem solving [5,6], develop more inclusive, participatory approaches to scientific inquiry, and generate innovative solutions to healthcare problems [7–9].

Human-centered design (HCD) training offers a promising toolset for researchers for enhancing the creativity and productivity of research teams by improving both team processes (e.g., communication, collaboration) and products (e.g., innovative and impactful science). HCD, a pragmatic approach to problem solving that draws from social science methodologies and is widely used in fields such as engineering and computer science, is “a flexible yet disciplined approach to innovation that prioritizes peopleʼs needs and concrete experiences in the design of complex systems” (p.1) [10]. It uses a systematic flow of well-structured activities designed to elevate voices that might not be otherwise heard, increase creativity, improve workflow processes, facilitate stakeholder buy-in, and generate more and better solutions to complex problems [11–16]. HCD is increasingly used in healthcare research to uncover unmet health needs [17–21], increase patient trust [22], design better interventions [23–31], and improve hospital space, workflows, processes, and policies [32–35]. However, the use of HCD to improve collaboration and innovation on scientific research teams is a more recent application, which, like much of the work on HCD in health, has not been rigorously evaluated [36]. In this paper, we investigate a unique initiative taken at the University of Pittsburgh focused on training clinical and translational researchers in the use of HCD in order to foster improved collaboration and greater innovation.

To evaluate this approach, we conducted a qualitative study with health sciences researchers (faculty and staff) who were trained in HCD methods. Our goals were to determine how these researchers applied HCD to their research, the benefits and drawbacks they perceived of applying HCD methods in their work, and the barriers and facilitators they perceived to using HCD.

## Methods

### Human-Centered Design Training

Between 2018 and 2019, the Clinical and Translational Science Institute (CTSI) at the University of Pittsburgh offered HCD training to a group of researchers from across the university. The goals of this training were twofold: (1) to help researchers develop techniques for drawing out and integrating the perspectives of all team members, thus improving collaboration and (2) to help researchers more creatively and empathetically engage stakeholders and team members in the design of interventions and processes, thus catalyzing more innovative problem solving. Training was provided by the LUMA Institute, an HCD design firm located in Pittsburgh with a highly regarded training program. LUMA has distilled the hundreds of design techniques contained within the general framework of HCD into 36 high-impact “methods” which can be combined into “recipes” to accomplish specific goals [37].

Three cohorts of trainees participated in the trainings in October 2018, May 2019, and October 2019. Trainees included 54 faculty and staff representing different roles on research teams, including principal investigators, coinvestigators, research coordinators, research facilitators, and community stakeholders. Whole teams were encouraged to participate together, though this was not always possible given scheduling challenges. Participants represented 20 departments and institutes across the Schools of the Health Sciences, with additional attendees from the schools of social work and engineering.

Training began with two in-person, full-day workshops conducted on the University of Pittsburgh campus. In these workshops, two experienced LUMA instructors led trainees through a set of structured activities, designed to introduce participants to the philosophy behind HCD as well as a range of HCD methods. The methods taught fell into three broad categories: (1) *looking* (methods for observing human experience, such as contextual inquiry and heuristic review); (2) *understanding* (methods for analyzing challenges and opportunities, such as abstraction laddering and stakeholder mapping); and (3) *making* (methods for envisioning future possibilities, such as creative matrixes and storyboarding). The methods, which often relied on flip chart paper and sticky notes of different colors, required participants to draw as well as write, and to be fully engaged with one another in pairs and small groups. The training approach provided participants with opportunities to see accomplished facilitators model each method while practicing it themselves.

This intensive training was combined with three follow-up sessions spaced over the following 3 months. For each session, groups of trainees (usually corresponding to an existing team) were asked to use one or more HCD methods in the context of their work and report back on their own experiences, receiving feedback from LUMA facilitators and other participants.

In addition to the 2-day training and follow-up sessions, participants received a booklet and a set of cards summarizing each of 36 high-impact HCD methods and 1 year of access to LUMA’s website, which offers training videos and tools for creating activities. The cost of the program to participants was covered by the University of Pittsburgh’s CTSI.

### Qualitative Data Collection

Of the 54 people who participated in HCD training, we contacted 41 to request interviews, excluding 13 who were either on or closely connected to the study team or who had left the university. Interviewees were informed that their participation was voluntary and confidential and were offered $50 gift cards in compensation for their time.

We conducted 1-hour, semi-structured qualitative interviews with all trainees who responded to our request. Interviews were conducted by a qualitative methodologist (MN) and a trained qualitative researcher from the Qualitative Evaluation and Stakeholder Engagement Research Service (Qual EASE). Interviews focused on trainee perceptions of the training, subsequent uses of HCD methods, barriers and facilitators to using HCD, and perceptions of the utility of HCD to science teams (see the Trainee Interview guide in Supplementary Table 1). Participants gave consent verbally before interviews were conducted. All research protocols were covered under IRB #0608202. It should be noted that, while HCD training was conducted face-to-face before the COVID-19 pandemic, interviews occurred after social distancing guidelines were in place, and were conducted via Zoom.

After interviews were transcribed verbatim and de-identified, data analysis was conducted by two experienced qualitative researchers (MN and MH), following the six-step process for thematic analysis described by Braun and Clarke [38]. A codebook (see Supplementary Table 2) was inductively developed by Drs. Norman and Hamm, who individually coded all the transcripts and adjudicated coding discrepancies to full agreement. Coding was done in NVivo 12 to facilitate data organization and analysis. Coded data were analyzed to identify themes in participant responses. These themes were discussed and refined with other team members who had not conducted or coded the interviews, but had experience with HCD methods. The results of the interview data are described later.

## Results

Of the 41 people we contacted, 18 responded to our request for interviews (9 faculty members and 9 staff members) and we interviewed them all. Faculty included both early and mid-career investigators and represented a broad array of fields, including Medicine, Pharmacy, Public Health, Engineering, and Social Work. Staff included individuals working in a wide range of research roles, including administration, study coordination, project management, recruitment, compliance, and community engagement. Staff also represented a broad spectrum of fields. From these interviews, we identified the following themes, which cut across faculty and staff:

### Theme 1: Participants Applied a Variety of HCD Methods in a Broad Range of Research-Related Contexts

Taken as a group, participants reported using a wide variety of HCD methods in their work, and doing so in a broad range of contexts, including stakeholder engagement, research design and project planning, and meeting facilitation and team management. There were variations in how intensively individual participants used HCD, however, with some using HCD methods periodically and others regularly. Specific ways participants applied HCD methods are summarized in Table 1.

**Table 1.** Applications of human-centered design methods

General areas of application	Specific applications
Stakeholder engagement and intervention design	Developing programs and interventions
	Identifying pain points for stakeholders
	Identifying barriers and facilitators to care
	Improving programs and interventions
	Providing information reciprocity to stakeholders
Research design and project planning	Brainstorming project ideas
	Identifying potential collaborations
	Writing innovation sections for grants
	Generating aims for grants
	Writing background sections for papers
	Writing discussion sections for manuscripts
	Evaluating and improving team processes
	Identifying obstacles to recruitment
	Prioritizing tasks
	Allocating resources
	Identifying goals and future projects
	Identifying possible publications
Meeting facilitation and team management	Promoting group cohesion/bonding
	Making team meetings more inclusive
	Making team meetings more participatory/engaging
	Soliciting the perspectives of all team members
	Improving group processes

#### Stakeholder engagement

Participants used HCD methods with stakeholders from different communities to explore experiences, determine needs, identify barriers and facilitators to care, and design or improve interventions. Participants reported using HCD methods to collect input from a wide range of research participant stakeholders both in the United States and in international research contexts. Applications included seeking input on the refinement of research questions, the creation of interventions, and collection of feedback on the experience of participating in research. Participants saw HCD as ideal for engaging stakeholders, particularly community members who might be less accustomed to or more distrustful of traditional research methodologies. As one participant said:

Human centered design tools, particularly for low-income or underserved populations or low-literacy groups that aren’t like academics…are so much more effective for quickly pulling out the main barriers and even facilitators to any type of problem. [Participant_1]

#### Research design and project planning

HCD methods were employed at multiple points in the process of planning and executing research projects. Many of the participants used HCD for idea generation, including to identify salient research questions and brainstorm project ideas. One intriguing application was the use of HCD to generate ideas for innovation sections of grants. As a participant noted, investigators often write the innovation section of grants themselves, but an HCD brainstorming exercise with the whole team could uncover far more ways in which a proposal was innovative. Teams also used HCD to brainstorm ideas for the discussion sections of manuscripts.

Participants found HCD methods useful for problem definition as well. One participant observed that while researchers often jump straight to fixing problems before they fully understand them, HCD methods can help teams interrogate whether the problem is important to solve in the first place or if the research question is the right one. Participants also used HCD methods to pinpoint potential sticking points in projects. One staff member, for instance, used HCD methods to identify potential risks to a project such as unrealistic recruitment targets, so they could be addressed early.

Participants also used HCD methods to organize and streamline information, collect feedback from differently positioned team members, set priorities, and allocate resources. As one individual told us:

Scientific teams are limited, funding is limited, people do multiple things, and they have to prioritize. If the investigator says it’s all important, it’s all critical, the team can be paralyzed.…Many of the [HCD] activities are prioritizing activities that can help you…decide what you want to do first. [Participant_17]

#### Meeting facilitation and team management

Participants used HCD activities to facilitate everything from weekly team meetings to departmental retreats to advisory board meetings. One participant commented that having structured HCD exercises for meetings reduced awkwardness and made him feel more confident as a leader. Several participants commented that their team meetings were more fun since they began using HCD methods and that attendance and participation at team meetings had improved.

Participants also used HCD methods to enhance communication and improve collaboration on their teams. Several participants regularly solicited input from their team members about team processes, to identify successes, challenges, and opportunities. For example, one participant used the “Rose Thorn, Bud” activity to check in with her team members and saved the sticky notes from these activities to show team members which “buds” (opportunities) had turned into “roses” (successes) by the end of the year. Others used sequences of HCD methods to ensure that the team shared the same vision for projects, using some methods to draw out individual goals and perspectives and others to build consensus around priorities and direction. As one participant described it:

[Using HCD], you can have the lab tech…the grad student, the post doc, the PI and maybe the co-investigator and it’s an opportunity to get them all in the room and…make sure that all the different thoughts and ideas they have [are aired], but everyone’s on the same page, too. [Participant_8]

### Theme 2: Participants Saw HCD Methods as Offering Clear Benefits for Researchers and Some Experienced HCD as a Fundamental Paradigm Shift

Participants overwhelmingly saw value in HCD methods for research teams. These benefits clustered in five areas: creativity, egalitarianism, structure, efficiency, and visibility. Representative quotes in each subtheme are provided in Supplementary Table 3.

#### Creativity

Participants generally believed that the highly participatory, hands-on nature of HCD activities engaged participants more deeply than traditional data collection and meeting facilitation methods and prompted out-of-the-box thinking and greater creativity. Participants credited HCD methods with helping researchers move away from linear thinking, examine their assumptions, bring more diverse voices to bear on problem solving, take more risks, and consider a broader range of ideas.

#### Egalitarianism

Another significant advantage of HCD methods, remarked on by many participants, was that it “levels the playing field” by giving every person involved in the exercise a voice and putting them on equal footing. Depending on the method, this was generally accomplished by providing time for individual thought before group discussion, asking everyone to write a specified number of ideas on sticky notes, and using structured methods of turn-taking. As one participant noted, “With [HCD], I feel like…everybody gets to move ideas around, everybody gets to write the same number of ideas, you know, everybody has input.” [Participant_01] Participants felt that HCD methods drew out the voices of differently positioned stakeholders and team members, and thus generated more potentially impactful ideas. The focus in HCD on generating ideas without judgment created a sense of safety, particularly for participants with less institutional power (e.g., staff and junior faculty), while the use of sticky notes promoted a feeling of anonymity that encouraged input. Several participants said they believed the egalitarian ethic of HCD also enhanced efficiency, since HCD methods both prevented a few individuals from dominating and potentially derailing discussion and encouraged early airing of concerns. One commented that in traditional research environments, staff often deferred to the PI, keeping doubts to themselves, and not raising concerns about project feasibility. They noted that by soliciting more input from staff early, HCD could potentially prevent time-consuming mistakes.

#### Structure

Many of the trainees talked about how the structured quality of HCD contributed to its effectiveness. Several participants remarked that giving the methods clear, memorable names (e.g., Round Robin, Statement Starters) helped to streamline communication on their teams. That methods could be combined into different recipes offered versatility within the structure. Participants appreciated how both the training and the materials provided by LUMA (including a booklet, methods cards, and an interactive website) helped them plan and facilitate HCD activities by providing detailed guidance on each method. One participant said: “They teach you how to do it…which really works for scientists; they want specific instructions” [Participant_17]. Many participants consulted these resources regularly before using HCD activities. The activities, moreover, provide what one trainee referred to as a “liberating structure” [Participant_15]: boundaries within which participants could be more creative.

#### Efficiency

A number of participants remarked on the potential of HCD to help research teams reach higher quality ideas more quickly. Most attributed this efficiency to the structure described above. Because each activity targeted a specific goal (e.g., brainstorming, prioritizing, ideating, collecting input) and was time-constrained, the methods discouraged long, rambling discussions, which, as one participant said, “allows you to get to the outcome faster” [Participant_12]. A number of participants believed HCD’s potential to reframe problems and prevent stale thinking also created efficiencies because it improved the quality of ideas a group could generate in the same or less time.

#### Visibility

To participants, the visual nature of some HCD methods (many of which rely on flip charts, sticky notes, drawing, storyboarding, and other visual representations) offered benefits over methods that do not bring the results of a data-gathering process into view. Participants reported that they were more accountable when using HCD methods because their collective thought process and decisions were displayed for all to see. Several also commented that having a physically tangible end product after an HCD activity was helpful for showing community partners the progress they had made during community engaged research. Several participants compared HCD with traditional methods such as focus groups and interviews in this regard. As one said: “When you do an interview with somebody or you do a focus group, it is really difficult for people to understand the ideas they have come up with…So it is so impactful when you just show people that we’ve done some things” [Participant_1]. A physically tangible end product was also viewed as fostering a more reciprocal process of cocreation with stakeholders.

While all the participants spoke positively about HCD, their response to the training varied in intensity. Some participants viewed HCD primarily as a complement to the quantitative skills they possessed. However, to a subset of participants, HCD represented a paradigm shift: an approach that felt like a long-awaited and natural fit for their personalities, values, and research goals. As one participant put it: “I realized that this training was kind of what I have been waiting for, for a large part of my career” [Participant_12]. These participants tended to use HCD methods regularly and in multiple contexts.

### Theme 3: Participants Identified a Number of Challenges to Facilitating HCD Activities Along with Ways in Which the Institution Could Provide More Robust Support for Sustained Use

While participants saw value in HCD methods and used them in a wide variety of contexts, they also encountered challenges applying them. Some of these barriers related to physical logistics, knowledge gaps, and issues of facilitation that participants felt could be overcome with additional institutional support. Others were deeper problems related to cultural mismatch and lack of buy-in.

#### Physical logistics

A number of HCD methods require supplies such as different colored sticky notes, markers, and large wall-sized sticky sheets of paper, as well as space to use them. Small meeting rooms dominated by a conference table make using these methods physically awkward. Additionally, the cost of the materials adds up and is not always provided for in departmental supplies or budgets, necessitating that the participant pay for them out-of-pocket.

Using shared meeting space can also pose a problem. One participant described successfully doing an HCD exercise but then having to rapidly vacate the conference room so that another group could come in and having to very quickly take all of the sticky notes off of the walls, losing some of the organizational structure in the process. These methods, while regarded as efficient for the generation of ideas, also do not easily fit into a standard half-hour or hour-long scheduled meeting time. Participants suggested that their institutions could help with these barriers by providing space and funding for HCD supplies.

#### Knowledge gaps

As comprehensive as the LUMA training was, participants noted gaps in their knowledge that made aspects of HCD facilitation challenging. Several expressed uncertainty about which methods to use in which situations, for instance, and said they wished that the training had included more examples from science to help them make these determinations. Others commented that they did not know what to do with the large amount of data HCD methods could produce. As one participant described it:

We had a ton of data at the end, so that was one of the challenges that I faced: “How do you work with and consolidate all of this data?” We learned about the methods and how to implement them but not necessarily how to analyze everything that we collect. (Participant_4)

Several participants with prior qualitative research or design experience wondered if the training provided sufficient understanding of HCD’s conceptual underpinnings. They wanted to make sure that trainees focused on the deeper purpose of HCD rather than the superficial and logistical attributes of specific methods. As one participant stated: “The [methods] are a means to an end. They are not the end” [Participant_14]. However, even participants who expressed these concerns appreciated the training and thought it provided a helpful tool set for colleagues who lacked formal qualitative training. For instance, one participant said:

I was trained as a qualitative researcher, and my first thought was this is kind of a poor man’s version of an academic discipline. But I’m much less critical having done it than I was beforehand. So obviously you can develop great qualitative research expertise [that goes beyond HCD] but not every clinical researcher has time to go get an anthropology degree, so I think [HCD] can be really useful. [Participant 17]

#### Issues of facilitation

Participants described several challenges related to facilitation of HCD methods, generally agreeing that the LUMA facilitators made it look easier than it was. Among the issues mentioned were that it was challenging to choose and sequence an appropriate set of activities to accomplish specific goals, explain and set up the activity, and facilitate the conversation. Participants suggested that having facilitation help from trained HCD practitioners would be beneficial. Indeed, several of them had received planning and facilitation assistance from an HCD-trained staff member in the CTSI and felt that they could not have done it without his assistance. Many also expressed a desire either for ongoing access to LUMA experts or for a “community of practice” – a collection of other people at the University using HCD methodologies to whom they could turn for advice and discussion.

It should be noted that HCD training was conducted face-to-face before the pandemic, yet interviews took place mid-pandemic. Thus, when we spoke to them, interviewees were encountering the challenges of facilitating HCD methods remotely. Interestingly, many of them embraced the challenge and were successfully using remote collaboration tools (e.g., Miro [39] and Mural [40]). Nevertheless, adapting methods to the remote environment and learning online tools was a logistical barrier that merits attention.

#### Mismatch

Participants frequently described a mismatch between HCD methods and the hierarchical, linear culture of academic science. The same creativity and egalitarianism that were regarded as strengths of HCD methods were also at odds with a scientific culture that operates on the assumption that a lab or project’s PI has “final say” in what happens on a project. As one staff member said, regarding whether or not HCD methods were used in their group:

Putting everyone in a room and [giving them] the same number of Post-it notes [for ideas] and so forth kind of removes those potential barriers. […] I think [HCD] brings to the table an equal opportunity for everyone…but at the end of the day those decisions are ultimately made by the person running that lab and that person is the one who has to decide whether they want to use these things. [Participant_8]

Another participant noted that “most of the people [in science and medicine] have been trained in kind of a lecture format, and there’s a level of comfort with that and there’s a level of discomfort in being asked to walk around the room, use a Post-it note” [Participant_17], indicating that the very participatory, hands-on nature of the methods themselves could be a mismatch for scientific culture.

#### Buy-in

Some participants experienced pushback on the use of HCD methods from departmental leadership who were unfamiliar with the methods, or who felt that they were unproven. One early-career investigator felt that they had taken a considerable risk “vesting my whole career and my K01 on this” [Participant_9] over departmental objections to the aim of their K award proposal that used HCD methods. Another participant described being treated scornfully by department leadership because they were so enthusiastic about the methods:

My department wanted nothing to do with this training. They thought I was a freak with my little markers and sharpies and Post-it notes. Like some of them made fun of me even. The problem was that I was totally sold on the value but my leadership was not. [Participant_12]

Others encountered resistance from peers who questioned using HCD methods in lieu of standard methods (asking, for instance: “Why can’t we just brainstorm?”) and from stakeholders unfamiliar with the approach. For instance, one participant told us:

Sometimes my participants are not receptive.…If people are never used to being asked their opinion and all of a sudden you want them to express their opinion…it’s not that they don’t know how to write on the Post-it notes, it’s that they don’t even know what to write because they had never been asked. [HCD] is a culture and when this culture is very different from the dominant culture you could run into issues. [Participant_15]

Overcoming lack of buy-in was particularly challenging for junior faculty and staff, who lacked the institutional standing to advocate for these methods.

## Discussion

Our findings show that HCD approaches are relevant and applicable to the work of clinical and translational research teams. Trainees used the HCD methods to accomplish a broad range of research-related tasks including identifying salient research problems, finding areas for collaboration, brainstorming project ideas, collecting feedback from differently positioned team members, setting priorities, and writing grants and manuscripts.

This study also suggests that the use of HCD methods on clinical and translational research teams has the potential to enhance collaboration and innovation in important ways. Participants in our study perceived HCD methods as beneficial for: (a) drawing out the perspectives of differently positioned team members and breaking down silos and hierarchies that can impede productive interdisciplinary collaboration; (b) generating a broad range of ideas, evaluating these ideas more systematically and effectively, and ultimately producing more informed and impactful research designs and interventions; and (c) engaging stakeholders across a wide range of projects, particularly stakeholders from demographic groups that might otherwise be skeptical about research. Indeed, our data suggest that the visual, tangible nature of HCD and the efficiencies provided by well-defined, time-limited activities serve the purposed of stakeholder engagement particularly well. These findings align with an emerging literature on the benefits of HCD for health research [13,36] as well as the focus in the science of team science on deliberately and systematically building strong skills in areas such as community engagement, knowledge sharing, and interdisciplinary communication [41–44]. At a time when interdisciplinary team science is proving essential for solving complex problems [1,2,4] and the need to bring diverse perspectives to bear on health issues is more obvious and pressing than ever [45], the ability of HCD methods to work against traditional hierarchies and solicit a broader range of voices is significant.

At the same time, our interviews indicate challenges to using HCD methods. These included logistical issues, such as requirements of space, time, and resources, that could prove to be an impediment to regular or successful implementation of HCD methods. Other challenges involved gaps in knowledge and confidence, specifically regarding sequencing activities and analyzing data. There were, moreover, situational challenges involved with having to move activities online during the COVID-19 pandemic. Finally, because HCD departs from the linear thinking and hierarchical norms typical of academia and science, there were challenges related to cultural mismatch and buy-in. We believe, however, that these challenges can be overcome. Logistical challenges can be met through the allocation of resources and space for HCD activities. Additional HCD training can mitigate knowledge gaps, provide needed practice opportunities, and help researchers adapt HCD methods for online implementation. The institution of communities of practice among HCD trainees and facilitators at or across institutions would provide avenues for researchers interested in HCD to get advice and support. Finally, strong institutional leadership in promoting and modeling HCD methods would help to generate buy-in.

Our study further suggests that providing HCD training directly to researchers is a model worthy of consideration. While other institutions have taken the approach of having HCD experts work with research teams [46,47], we believe that HCD training for researchers is beneficial in its own right, serving different researchers in different ways. Some, like the “power users” among our trainees, may develop an immediate affinity for HCD that prompts them to use the methods intensively, pursue additional training, develop expertise of their own, and become institutional “influencers.” Others might gain sufficient confidence to use simpler HCD methods but may need help applying more complex ones. Still others may never become facile with HCD methods but would nevertheless gain an understanding from the training that would allow them to work more effectively with HCD experts, in much the same way as researchers with some statistical or qualitative training are better able to work more effectively with statisticians and qualitative methodologists. To address these levels of engagement, one potentially productive model would be for institutions to provide HCD training to researchers *and* cultivate a core of trained HCD facilitators who can work with researchers to select, plan, if necessary, cofacilitate or facilitate HCD activities.

Taken together, we believe institutions interested in bringing HCD into team science training should be heartened by the findings from this research, which suggests that HCD training is both viewed as useful and relevant to research teams and helpful for improving collaboration and innovation. However, interested institutions would also do well to recognize that the effective integration of HCD requires not only initial training for researchers but also ongoing support and an institutional commitment to making HCD part of the culture.

There were a number of limitations to this study. Since interviewees participated in LUMA training in three cohorts over 2 years, the training was fresher in the minds of some interviewees than others. Our sample was limited by the available pool of LUMA trainees and further constrained by those who agreed to be interviewed. Additionally, interviews were conducted in the early days of the COVID-19 pandemic, which may have decreased the number of participants willing to be interviewed. It may also have influenced their perspectives. Interview data reflect the subjective experience and recollections of individuals. Thus, it serves as a proxy for actual behavior, not an objective measure of that behavior. Finally, while a qualitative study exploring trainee perspectives on and uses of HCD is an appropriate first step, other methodologies (e.g., observational studies, a longitudinal grant or manuscript review) would be required to evaluate whether HCD training resulted in measurable changes in the quality of team collaboration and innovation. These would be fruitful opportunities for future research.

In light of this study, we plan to expand access to HCD training for researchers, develop a core of trained HCD facilitators who can cofacilitate activities and provide in-house training, identify HCD methods with particular salience to clinical and translational researchers, develop training examples and models customized to our own professional contexts, and foster communities of practice to provide peer–peer support among researchers employing HCD methods.

## Conclusion

HCD is gaining adherents in biomedical and translational research [16,21,32,33], yet until recently, there has been little documented evidence to validate its use. This study provides empirical data to support the contention that researchers trained in HCD do, in fact, use these methods and find them useful for accomplishing a broad range of tasks. The benefits participants cite from using HCD methods suggest that HCD has the potential both to help researchers work more inclusively and collaboratively on interdisciplinary teams and generate more creative and innovative science. It also suggests that robust training and sustained institutional support will be needed to help researchers use HCD to its full potential.

## Supplementary material

For supplementary material accompanying this paper visit https://doi.org/10.1017/cts.2021.794. click here to view supplementary material
