# Supplementary material for: Assessing the application of human-centered design to translational research
Source: J Clin Transl Sci. 2021 May 24;5(1):e130. doi: 10.1017/cts.2021.794 (PMC8327548; doi:10.1017/cts.2021.794)
Supplement: Supplementary file 1 [file S2059866121007949sup001.docx]

**Supplementary Table 1: Interview Guide**

1. How did you come to participate in LUMA training?
   1. Did you have any previous HCD or design training or experience?
   2. If so, what was it?

In this interview, we’re interested in learning about three things: your experience at the 2-day training, your experience in the follow-up sessions, and how (if at all) you’ve used HCD methods since completing the training.

1. First, let’s talk about your perceptions of the LUMA 2-day training...
   1. What did you find most/least useful about the training?
   2. Was there anything you found surprising?
2. Next, let’s talk about the follow-up sessions…
   1. How many did you attend? If none, what prevented you?
   2. What did you find most/least useful about these sessions?
   3. Was there anything you found surprising?
3. Finally, have you used HCD methods since the training ended?
   1. (If yes)
      1. Which methods did you use?
      2. How did you use them?
      3. With whom?
      4. What advantages did you see to using these methods?
      5. Could you have achieved the same goals using other means? Explain.
      6. What challenges did you encounter?
      7. Is there anything that would help you use HCD methods (e.g., additional training or resources, having a trained facilitator help you run HCD sessions)?
      8. Are there ways you can imagine using HCD methods in the future?
   2. (If no)
      1. Are there reasons you haven’t used them?
      2. Is there anything that would help you use them (e.g., additional training or resources, having a trained HCD facilitator help you run HCD sessions)?
      3. Are there ways you can imagine using HCD methods in the future?
4. Has HCD training made you think differently about working on a science team? Explain.
5. Think about a typical science team and the work it needs to accomplish (e.g., brainstorming ideas, identifying aims, writing innovative proposals, collaborating, managing projects...) What do you think HCD offers that would help teams achieve these goals?
   1. Orientation/approach
   2. Specific methods? How might you use them?
   3. Who on the team would benefit most from learning HCD methods?
6. Anything you’d like to add? Anything we should have asked but didn’t?

**Supplementary Table 2: Codebook**

| CATEGORY | CODE | DESCRIPTION |
| --- | --- | --- |
| ENTRY POINT | ENTRY_HOW | Describes how trainees got involved in LUMA training, including who told them about the training, invited them, or covered the costs |
|  | ENTRY_WHY | Offers specific personal or professional reasons for gaining HCD skills, and/or talked about what they hoped to gain by participating |
|  | ENTRY_PREV_HCD | Describes any previous experience with or knowledge of Human Centered Design or of LUMA |
| 2-DAY TRAINING | TRAIN_POS | Describes positive aspects of the initial, 2-day training, e.g., meeting people from across campus, good facilitation |
|  | TRAIN_NEG | Describes negative aspects of the initial, 2-day training, e.g., exhausting, too long, hard to work around schedule |
|  | TRAIN_SUGGEST | Offers suggestions for improving initial, 2-day training, e.g., provide more examples from research, spread out over more time |
| FOLLOW-UPS | FUPS_POS | Describes positive aspects of the three follow-up sessions, e.g., hearing ideas from colleagues, discussing applications |
|  | FUPS_NEG | Describes negative aspects of the three follow-up sessions, e.g., hard to schedule, not helpful to hear how others applied HCD methods |
|  | FUPS_SUGGEST | Offers suggestions for improving the follow-up sessions, e.g., schedule farther apart, give groups more time to present, include lunch |
|  | FUPS_DIDN’T_ATTEND | Explains that and/or why they did not attend one or more follow-up sessions, e.g., couldn’t fit into schedule, found out too late |
| APPLICATION | APPLY_FACILITATORS | Describes factors that made use of HCD methods possible or contributed to trainees’ comfort level, e.g., facilitation help, practice |
|  | APPLY_BARRIERS | Describes specific factors that inhibited or discouraged use of HCD methods (e.g., lack of confidence/materials) |
|  | APPLY_METHODS | Mentions (by name or description) specific methods used, e.g., Rose-Thorn-Bud, Creative Matrix, Storyboarding |
|  | APPLY_ADAPT | Describes adapting HCD methods to new contexts or use cases, including approaches and experiences (positive and negative) |
| USES | USES_ACAD | Describes the uses of HCD methods in the context of their academic or professional work, e.g., team meetings, advisory boards, work teams |
|  | USES_NON-ACAD | Describes the uses of HCD methods outside the context of their academic or professional work, e.g., neighborhood group, PTA, family |
|  | USES_GOALS | Describes specific goals they have had when using HCD methods, e.g., critiquing specific aims, getting feedback on an app. |
| HCD_POS | HCD_POS_IDEAS | Describes the ability to generate more or better ideas or solutions by using HCD methods. |
|  | HCD_POS_CREATIVITY | Describes approaching tasks or problems with greater inventiveness. thinking outside the box |
|  | HCD_POS_EFFICIENT | Describes HCD as saving time, preventing time-consuming errors, or providing better solutions faster |
|  | HCD_POS_EGAL | Describes getting input from all team members, a more equitable environment, breaking down power hierarchy using HCD methods |
|  | HCD_POS_SAFE | Describes feeling like participants can offer ideas and perspectives openly, without feeling critiqued or judged when using HCD methods |
|  | HCD_POS_INSIGHT | Describes gaining new insight into problems, processes, or team dynamics using HCD methods |
|  | HCD_POS_EGAL | Describes getting input from all team members, a more equitable environment, breaking down power hierarchy using HCD methods |
|  | HCD_POS_VISIBILITY | Describes advantages of the visual nature of HCD for being able to see/share work or results |
|  | HCD_POS_FLEXIBILITY | Describes the applicability or versatility of HCD methods for a variety of uses and contexts |
|  | HCD_POS_ENGAGE | Describes sense that HCD methods compelled more active or full participation |
|  | HCD_POS_FUN | Described HCD methods as enjoyable, engaging, energizing, or novel |
|  | HCD_POS_IMPACTFUL | Describes HCD as generating ideas that were ultimately more relevant or useful for individuals and/or communities |
|  | HCD_POS_OTHER | Describes benefits of using HCD methods other than the ones described by other codes |
| HCD_CHALL | HCD_CHALL_MISMATCH | Describes misalignment or mismatch of HCD methods to culture of academic medicine or science (e.g., non-linear, playful, exploratory) |
|  | HCD_CHALL_UNFAMILIAR | Describes challenges stemming from participants’ lack of experience with or exposure to HCD methods |
|  | HCD_CHALL_LOGISTICS | Describes challenges having to do with resources (e.g., stickies, pens, flipcharts) and space |
|  | HCD_CHALL_ANALYSIS | Describes not knowing how to organize or process data produced using HCD methods |
|  | HCD_CHALL_TIME | Describes challenges involving the time required to plan, prepare for, and use HCD methods |
|  | HCD_CHALL_BUY-IN | Describes having trouble getting buy-in from participants regarding the use of HCD methods (too touchy-feely, too time-consuming) |
|  | HCD_CHALL_PRESSURE | Describes feeling pressure to participate actively and/or pressure to come up with ideas |
|  | HCD_CHALL_SAFETY | Describes concerns about lack of trust within group or an insufficiently safe environment for speaking honestly |
|  | HCD_CHALL_OTHER | Describes challenges of using HCD methods other than those included in other codes. |
| COMPARISON | ADVANTAGES_COMPARE | Describes advantages of using HCD methods in direct comparison to traditional or alternative methods, e.g., higher quality solutions |
|  | DISADVANTAGES_COMPARE | Describes disadvantages of using HCD methods in direct comparison to traditional or alternative methods |
| LUMA | LUMA_FACILITATORS | Reference to approach or quality of LUMA facilitators for training and follow-up sessions |
|  | LUMA_WORKPLACE | Reference to LUMA website/”Workplace” and any materials provided digitally |
|  | LUMA_MATERIALS | Reference to LUMA print materials, including cards, booklets, stickers, and anything not provided digitally |
|  | LUMA_QUESTIONS | Raises specific questions about LUMA, e.g., whether methods are proprietary, who can use/teach them. |
|  | LUMA_SUGGESTIONS | Offers specific suggestions to LUMA that go beyond training and follow-ups (e.g., development of a phone app, starter pack of supplies) |
| CTSI | CTSI_SUPPORT/FACIL | Specific reference to useful resources or support provided by CTSI (Pitt), including facilitation help |
|  | CTSI_SUGGESTIONS | Offers specific suggestions to CTSI that go beyond offering training (e.g., development of a community of learners, budget for materials…) |
| HCD_ONLINE | HCD_ONLINE_APPLY | References to applying HCD methods virtually, whether already attempted or imagined, including affordances and challenges |
|  | HCD_ONLINE_TOOLS | References to tools or technologies (e.g., Mural) for applying HCD methods virtually, whether already attempted or imagined |
| HCD_TS | HCD_TS_APPLICABILITY | Describes general applicability of HCD methods to science teams and their typical tasks (e.g., grant writing, manuscript writing) |
|  | HCD_TS_THINKING | Describes whether or not HCD has changed their thinking about science teams and, if so, how |

**Supplementary Table 3: Benefits of HCD as identified by participants with representative quotes**

| Sub-Theme | Representative Quotes |
| --- | --- |
| Creativity | The science world is very fact-based and there’s a process for everything. [HCD] is a process, but I think it’s more of a creative process. So I think that does kind of force some of the more technical folks to open up a little bit more, and I think that it’s helpful. [Participant_05]  You know, [typical scientists] just sit there and talk [using] business-as-usual types of methods, then say: okay we talked about it for ten minutes so let’s go ahead, write our paper, and get it published in Nature [laugh]. I mean, there is nothing wrong with publishing a paper in Nature; that’s great. But how many assumptions did we really examine? How much creative tension did we really engage in? I think LUMA methods provide…a novel method for scientists to put them on edge and predispose them to think differently and [make them] open to new ideas and exploration. [Participant_15]  Oftentimes in a regular team meeting, [you] might just sit around a table and talk, whereas I feel like [HCD] methods encourage people to come up with creative, off-the-wall ideas which is really helpful. [Participant_13] |
| Egalitarianism | I think that it allows more equality across the team and gives everyone a voice. Even if they are like a first-year undergrad, they still have an opportunity to share their ideas…[Participant_13]  So these methods are useful because they equalize the playing field, right? They sort of help each idea have the same amount of weight regardless of who it came from and they help with people monopolizing the conversation. [Participant_10]  A great advantage [of HCD] is that you can get feedback from a wider group of people and that hopefully will lead to a better working product. Stakeholders are important and their opinions are important. If you don’t consider [their feedback] then you walk away with something that people aren’t going to use. [Participant_009] |
| Structure | There’s something about the way that it’s packaged together that allows you to get to the outcome faster. [Participant_012]  [HCD methods] can really help to set a base line for communication conventions that will further a team’s ability to function cohesively. [Participant_2]  So many times I’ve been to meetings where, if there are 20 people, only 4-5 people talk 90% of the time and you just can’t shut them up. But if you create the right structure then they wouldn’t be able to do that. [Participant_15] |
| Efficiency | “It should make your work more impactful because you’re spending less time running in circles and more time getting to the heart of what you need to do.” [Participant_1]  [HCD] may push people to think a little bit differently. Someone said: ‘If all you have is a hammer, everything looks like a nail.’ Well, I think that what LUMA does is it sort of takes the hammer out of your hands. It lets you say, you know, let’s look at this problem in a variety of ways. So I think it gets you [to a solution] sooner. [Participant_12]  [HCD methods] are time limited. You could get a bunch of community members together to talk about gun violence, let’s say, and that community might have a really meaningful conversation and not come up with any actual solutions in a two or three-hour meeting. With two or three hours of human-centered design methods you could definitely come up with several concrete solutions. You might not necessarily have all of the steps to implement each of those solutions, but it’s more likely that you’re gonna get closer to where you actually wanted to be. [Participant_10] |
| Visibility | One thing is that when you do an interview with somebody or you do a focus group, it is really difficult for people to understand the ideas they have come up with while they’re talking about them, right? So it is so impactful when you just show people on the board what they thought was important. [Participant_1]  I do think there’s something about putting everything up on a big wall that really helps you see what’s important. [find source]  Rather than just having a conversation and hoping someone captures it in notes or that we have recordings that we don’t revisit, [the Post-its and sticky sheets] serve as a document that synthesizes items to act on. [Participant_3] |
